# Inactivation kinetics of selected pathogenic and non-pathogenic bacteria by aqueous ozone to validate minimum usage in purified water

**DOI:** 10.3389/fmicb.2023.1258381

**Published:** 2024-01-15

**Authors:** Yuqian Lou, David R. Kasler, Zach L. Hawkins, Zhen Li, Dan Sannito, Ronald D. Fritz, Ahmed E. Yousef

**Affiliations:** ^1^PepsiCo R&D, Valhalla, NY, United States; ^2^Department of Food Science and Technology, The Ohio State University, Columbus, OH, United States; ^3^InSite Technologies, Inc., Burnsville, NC, United States; ^4^Department of Microbiology, The Ohio State University, Columbus, OH, United States

**Keywords:** purified water, ozone, pathogenic bacteria, Ct value, data modeling, heteroscedasticity, *Enterococcus faecium*, water pathogen surrogate

## Abstract

Ozone is often used as an antimicrobial agent at the final step in purified water processing. When used in purified bottled water manufacturing, residual ozone should not exceed 0.4 mg/L, per US-FDA regulations. These regulations require the control of *Escherichia coli* and other coliform bacteria; however, non-coliform pathogens can contaminate bottled water. Hence, it is prudent to test the efficacy of ozone against such pathogens to determine if the regulated ozone level adequately ensures the safety of the product. Inactivation of selected pathogenic and non-pathogenic bacteria in purified water was investigated as a function of ozone dose, expressed in Ct units (mg O_3_*min/L). Bacterial species tested were *Enterococcus faecium*, *E. coli* (two serotypes), *Listeria monocytogenes* (three strains), *Pseudomonas aeruginosa*, and *Salmonella enterica* (three serovars). Resulting dose (Ct)-response (reduction in populations’ log_10_ CFU/mL) relationships were mostly linear with obvious heteroscedasticity. This heteroscedastic relationship required developing a novel statistical approach to analyze these data so that the lower bound of the dose-response relationships can be determined and appropriate predictive models for such a bound can be formulated. An example of this analysis was determining the 95%-confidence lower bound equation for the pooled dose-responses of all tested species; the model can be presented as follows: *Logpopulationreduction* = 3.80*Ct* + 1.84. Based on this relationship, application ozone at a Ct of 0.832 and 21°C achieves ≥ 5-log reduction in the population of any of the tested pathogenic and non-pathogenic bacteria. This dose can be implemented by applying ozone at 0.832 mg/L for 1 min, 0.416 mg/L for 2 min, or other combinations. The study also proved the suitability of *E. faecium* ATCC 8459 as a surrogate strain for the pathogens tested in the current study for validating water decontamination processes by ozone. In conclusion, the study findings can be usefully implemented in processing validation of purified water and possibly other water types.

## 1 Introduction

Drinking water has been associated with numerous disease outbreaks, and the waterborne disease risk is increasing for many reasons. According to a recent estimate of the burden of waterborne diseases in the US, 7.15 million illnesses occur annually, and this results in 118,000 hospitalizations and 6,630 deaths each year (Collier et al., 2021). Pathogens often responsible for waterborne diseases include *Cryptosporidium* (Gharpure et al., 2019), *Giardia* spp. (Conners et al., 2021), Shiga toxin producing *Escherichia coli* (Olsen et al., 2002; Hrudey et al., 2003), *Pseudomonas aeruginosa* (Mena and Gerba, 2009), *Helicobacter pylori* (Aziz et al., 2015), *Vibrio* spp. (Shah et al., 2012), and noroviruses (Jacqueline et al., 2022). Water purification, as described later, ideally removes microbial contaminants, and purified or even non-purified water is bottled, depending on the prevailing regulations in different regions of the world. Purified water is often made from public sources of drinking water. If pathogenic and nonpathogenic microorganisms, originating from these public sources, find their way to purified water, they may survive or even grow in this environment (Warburton et al., 1998; McAlister et al., 2002; Djaouda et al., 2020). Additionally, contaminates may gain entrance during bottling, which is typically a non-aseptic process, or be present in bottles or caps prior to filling, particularly if bottles are formed outside the bottling plant and need to be shipped in for filling (Ramalho et al., 2001).

For ensuring water quality and safety in the United States, drinking water is regulated by the Environmental Protection Agency (EPA), whereas bottled and bottled-purified waters are regulated by the Food and Drug Administration (FDA). Bottled water simply is potable water that is sealed in bottles or other containers, and it may contain safe and suitable antimicrobial agents (Code of Federal Regulations, 2023c). Purified water is prepared from water complying with the regional water regulations or international guidelines, such as the US-EPA national primary drinking water regulations, the European Union or Japan drinking water regulations, or the World Health Organization’s guidelines for drinking water (United States Pharmacopeia, 2011). To achieve purified water’s acceptable safety and quality, drinking water is subjected to de-chlorination, reverse osmosis, distillation, deionization, ultraviolet radiation, ozone, or combinations of these processes (Rosenburg, 2003; United States Pharmacopeia, 2018). Bottled purified water is simply a packaged purified water and it should meet both FDA regulations for bottled water and the United States Pharmacopeia (USP) requirements for purified water. Purification steps are incorporated into purified bottled water manufacturing; hence, the product is low in total dissolved solids and inorganic and organic matters. Such product, therefore, has low ozone demand, which depends on the amount of oxidizable material in the water. Consequently, the efficacy of ozone against bacteria in purified water is expected to be greater than that in other types of drinking waters.

In commercial production of bottled purified water, ozone has been widely used. According to FDA regulations, residual ozone level at the time of bottling should not exceed 0.4 mg ozone per liter of bottled water (Code of Federal Regulations, 2023a). In addition, aqueous ozone has been approved in the US, at a minimum of 0.1 mg/L level, for use to sanitize water-contact surfaces and other critical areas in water bottling facilities (Code of Federal Regulations, 2023b). Hence, ozone now is commonly used in water purification facilities to sanitize product lines and package materials, to control the microbial quality of purified water in storage tanks, and to inactivate microbial contaminants on bottle fillers. The ozone dose applied during water treatment is often expressed as Ct, which is the multiplication product of aqueous ozone concentration (C) in mg/L, and ozone reaction or contact time (t) in minutes; therefore, Ct unit is mg*min/L. It is common to define the ozone dose (Ct value) required to achieve a certain level of disinfection against a targeted pathogen at a given temperature. EPA, for example, considers an ozone Ct value of 1.43 mg*min/L sufficient to cause a 3-log reduction in *Giardia* cysts population at 10°C (EPA, 2020). In comparison, using free chlorine to achieve the same lethality under similar conditions, a Ct value of 73–292 mg*min/L is needed, with value variations depending on medium pH and the chlorine concentration applied.

The 1982 regulatory approval of ozone use as a disinfectant in bottled water, at a maximum level of 0.4 mg/L at the time of bottling (Food and Drug Administration, 1982), was based on ozone usage data and published efficacy studies at the time of petition submission. However, there is a lack of ozone efficacy data in purified water, which is very low in total solids and ozone demand; hence, there is a need to re-examine the level of ozone usage in purified water, in terms of efficacy against bacterial pathogens. With these considerations in mind, the current research was initiated to study the inactivation of bacteria, relevant to purified water manufacturing, as a function of ozone dose, expressed in Ct units, and expressing the results in dose-response functions. Unlike some previously published research, low-count cell suspensions were applied to simulate realistic contamination levels of purified water, and to minimize the organic load imposed by the high cell populations. The investigation covered pathogenic and non-pathogenic bacteria, and a strain that may serve as a pathogen surrogate. The kinetic study should allow defining the ozone doses required to achieve the safety of purified water.

## 2 Materials and methods

### 2.1 Culture and cell suspension preparation

Ten strains of five bacterial species were investigated in the current study (Table 1). Each strain was transferred from its frozen stock (at −80°C in 15% glycerol-containing media) by streaking onto tryptic soy agar (TSA; Becton Dickinson, Sparks, MD, USA) and incubating streaked plates for 16–24 h at 35°C. An isolated colony of each strain was transferred from a TSA plate to tryptic soy broth (TSB; Becton Dickinson) and incubated overnight at 35°C; this incubation was followed by a second transfer in TSB and incubation as described for the previous transfer. Subsequently, 1 mL of the resulting culture was centrifuged at 4°C and 4,500 × *g* for 8 min and the cell pellet was washed twice using 1 mL aliquots of sterile 0.85% NaCl (saline) solution. The washed pellet was resuspended in 1 mL of saline solution and decimally diluted (in saline solution) to the level needed for each prepared inoculum. Targeted inoculum to be added to the 100-mL reaction volume was 10^6^–10^7^ CFU/mL, whereas the inoculum prepared to be added to the 1,000-mL reaction volume was 10^7^–10^8^ CFU/mL; therefore, both reaction volumes contained 10^4^–10^5^ CFU/mL.

**TABLE 1:** Bacterial strains tested in the current study.

Strain	Original source	References
*Enterococcus faecium* ATCC 8459 (NRRL B-2354)	Unknown	Kopit et al., 2014; https://www.ncbi.nlm.nih.gov/Taxonomy/Browser/wwwtax.cgi?id=1104325
*Escherichia coli* K12 (ATCC 10798)	Stool sample	Dimitrova et al., 2017
*E. coli* O157:H7 B6914	Stool sample	Uhlich et al., 2017
*Listeria monocytogenes* California	Mexican-style cheese	Ryser and Marth, 1987
*L. monocytogenes* Scott A	Clinical isolate	Ryser and Marth, 1987
*L. monocytogenes* V7	Milk isolate	Ryser and Marth, 1987
*Pseudomonas aeruginosa* PRD-10 (ATCC 15442)	Water bottle in an animal room	https://www.atcc.org/products/15442
*Salmonella enterica* ser. Livingstone 1236H	Peanut butter	Peña-Meléndez et al., 2014; Abdelhamid et al., 2021
*S. enterica* ser. Tennessee E2007000304	Peanut butter	Enache et al., 2015
*S. enterica* ser. Typhimurium LT2 (ATCC 700720)	Natural source	https://www.atcc.org/products/700720d-5

### 2.2 Purified water

Purified water of different sources was tested in this study. Commercial bottled purified water (Aquafina; PepsiCo, Inc., Purchase, NY, USA) was used. The water from this source has the following characteristics: conductivity, 6.02 μs/cm; total dissolved solids, ≤10 mg/mL; pH 5.5–7.0. Additionally, deionized municipal water (Columbus, OH, USA) was used in some experiments. The deionized water had the following characteristics: conductivity, 1.03 μs/cm; total dissolved solids, ≤10 mg/mL; pH 6.5–8.0.

### 2.3 Glassware

To ensure total removal of all biological material that may interfere with ozone, all glassware used in these experiments was washed with detergent and tap water, thoroughly rinsed with deionized water, and sterilized via autoclave for 20 min at 121°C prior to use. As a final control measure for residual ozone-demanding material, the flasks used for ozone-bacteria reactions were also rinsed with water containing 0.5 mg/L ozone.

### 2.4 Experimental procedure

**FIGURE 1:**
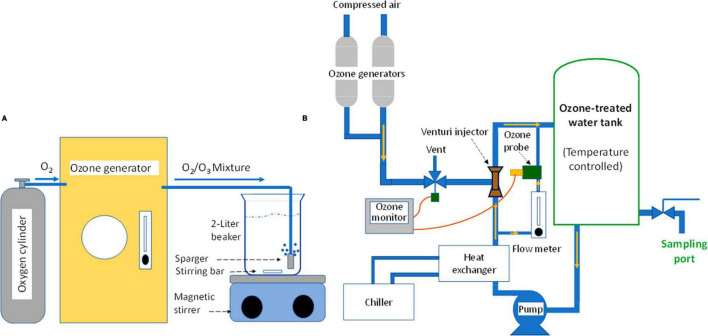
Devices used for production of ozone-treated water, which was tested in the current study. [Setup **A**]: Bench-top unit, targeting 1.5 mg/L or lower ozone in water; [Setup **B**]: Mini-ozone skid, targeting 0.05–1.2 mg/L ozone in water.

**FIGURE 2:**
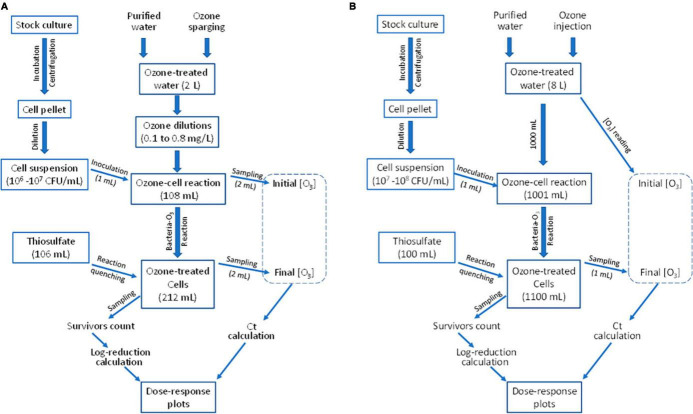
Procedures for determining microbial inactivation in response to ozone dose to develop the dose-response relationship. **(A,B)** Procedures completed using Setup A and Setup B of Figure 1, respectively.

The ozone-bacteria reaction experiments were conducted using one of two equipment setups, depending on the desired ozone concentration range and the sample volume needed to achieve desired minimum detection level of treatment survivors. In Setup A, 2 L of purified water was ozone-treated using a bench-top ozone generator (Figure 1A) and 100 mL of aqueous ozone-cell suspension reaction mixture was tested (Figure 2A). In Setup B, ∼8 L of purified water was treated with ozone using a custom-designed mini-ozone skid (Figure 1B). In this setup, 1,000 mL of aqueous ozone-cell suspension reaction mixture was tested (Figure 2B).

#### 2.4.1 Setup A

##### 2.4.1.1 Ozone production

The ozone equipment used in Setup A is shown schematically in Figure 1A. An ozone generator (Model LG-14; Dell Ozone, San Luis Obispo, CA, USA) was used to produce ozone from compressed oxygen at a maximum of 14 g O_3_/h. Ozone generator settings were based on the desired ozone level for a given experiment, which was between 0.8 and 1.5 mg/L in the stock ozone solution. The ozone gas stream was bubbled, through a sparger, into a 2-L beaker filled with purified water while mixing with a stirring bar. Bubbling lasted for at least 10 min to reach equilibrium; this was verified by measuring ozone using the Indigo method, as described later. If concentration deviated from the expected value, small adjustments of the generator power setting were made and retested. Once at the desired level, samples of ozone-treated water were transferred using sterile glass pipettes or graduated cylinders into reaction flasks and proper ozone dilutions were made. The generator was left running during the entire time of testing to maintain the equilibrium ozone concentration during the day’s experimental runs.

##### 2.4.1.2 Ozone-bacteria reactions

A flow chart (Figure 2A) shows the experimental procedure used in Setup A. Purified water was dispended into sterile 250-mL flasks containing magnetic bars and placed on magnetic stirrers (Corning PC 351, Corning, NY, USA), with volumes of 0, 54.5, 81.8, and 95.4 mL added to these flasks. Measured volumes of ozone-treated water were added to its respective flask to a total volume of 109 mL per flask. These volume ratios were determined to achieve different desired ozone concentrations. Upon addition of ozone-treated water having 0.80 mg/L, these volume ratios yielded 0.80, 0.40, 0.20, and 0.11 mg/L ozone, respectively. Upon a quick mixing, two mL of each O_3_-H_2_O mixture was quickly pipetted out (leaving 107 mL per flask) to be used in measuring the initial ozone concentration using the Indigo method. Immediately after, 1 mL of the 10^6^–10^7^ CFU/mL cell suspension was added to each flask for a total volume of 108 mL/flask. After 10–90 s of reaction, depending on the desired Ct value, 2 mL of the reaction mixture was pipetted out for the second ozone measurement. Ozone reaction in the remaining reaction mixture (106 mL/flask) was quenched using 106 mL of pre-prepared sodium thiosulfate solution (Na_2_S_2_O_3_), 0.12%, for a total volume of 212 mL per flask. The flask contents were mixed for 15 s to ensure adequate quenching. Use of sodium thiosulfate was not found to cause any additional microbial inactivation (data not shown). Other researchers similarly used thiosulfates as a disinfectant reaction quencher (Kemp and Schneider, 2000; Azuma et al., 2022).

Next, cells in 200 mL of the flask contents were harvested using a filter-plate kit. These 200 mL were pipetted through the microfilter (Cat. No. 6550; Neogen, Lansing, MI, USA), followed by dispensing a pre-prepared 2 mL media ampoule (Cat. No. 6516; Neogen) containing membrane tryptone glucose extract (m-TGE) broth with a viable-cell-stain indicator. Additionally, 1 mL of the remaining volume in the flask was spread-plated across four TSA plates (250 μL, each). Lastly, 0.1 mL of flask contents was spread on a single TSA plate. This sampling scheme gave three levels of detection: small population detected on the filter-medium kit, medium population on the 1-mL spread plates, and high population on the plate receiving 0.1 mL of reaction mixture. The untreated control was prepared by combining 107 mL of non-ozone-treated water with 1 mL of cell suspension and 108 mL of sodium thiosulfate. Dilutions were prepared of this control sample and 0.1 mL of the 10^0^, 10^–1^, and 10^–2^ dilutions were spread-plated on TSA in duplicate plates. All plates were incubated at 35°C for 48 h before colonies were enumerated.

#### 2.4.2 Setup B

##### 2.4.2.1 Ozone production

For ozone generation in Setup B, a custom-built “mini-ozone skid” was used for making ozonated water (Figure 1B). This equipment made it possible to test ozone concentrations that were not achievable by Setup A. The skid featured a 10-liter stainless-steel reactor tank (La Nuova Sansone, Lecce, Italy). The tank holds the water to be ozone-treated and it is equipped with a sampling port for sample dispensing and a temperature sensor (IFM, Malvern, PA, USA). The skid was also equipped with two ozone generators (Oxidation Technologies, LLC Inwood, IA, USA), which use air to generate ozone at two capacities, 200 mg O_3_/h (OZX-300AT; Oxidation Technologies) and 4 g O_3_/h (Model VMUS-4; Oxidation Technologies). The small ozone generator was used for targeted aqueous ozone of ≤1 mg/L and the larger unit would be required for ozone level > 1 mg/L. A Venturi injector (Mazzei injectors, Bakersfield, CA, USA) was used for incorporating ozone gas into the water circulation loop and the air/ozone gas mixture flow rate was controlled by a needle valve and a solenoid valve (Parker, Cleveland, OH, USA). The skid also included a pump (Model 72021-32; Cole-Parmer, Vernon Hills, IL, USA) for circulating the ozone-treated water, a chiller (H50-500; Labtech, Hopkinton, MA, USA) for controlling water temperature, and ozone concentration display and control panel (Rosemount 499AOZ Dissolved Ozone Sensor; Emerson Electric Co., Ferguson, MO, USA), which displays ozone concentration and water temperature, and allows for adjusting the ozone concentration setpoint by controlling the gas-flow solenoid valve. A sensor was installed in the water circulation loop and was used to measure ozone concentration (mg/L) in the water tank. Water flow through the sensor was regulated at 5 gph using a float-type flow meter (7520/7530 Series; King Instrument Company Inc., Garden Grove, CA, USA). The sensor reading was continuously displayed on the unit display and the control panel. The mini-ozone skid was also equipped with a cleaning-in-place (CIP) loop for equipment cleaning and sanitizing, if needed.

For operation of the mini-ozone skid, the tank was filled with purified water and circulation started. After a few minutes of circulation, the reading of the ozone monitor was adjusted to zero. The generator was then turned on and programmed to the desired ozone level. The chiller was set to maintain 21°C and the system was allowed to run for 5 to 10 min to reach the equilibrium. Ozone concentration was then verified using a commercial ozone measuring kit as described later. If the ozone reading deviated from the desired value, the skid controller was readjusted, and the system was retested. Once set to the desired ozone concentration and the equilibrium point was reached, the 1,000-mL sample was dispended from the sample port into a 1,000-mL flask. Unlike the 100-mL reactions described earlier (Setup A), no dilution of the prepared ozone solutions was performed. During the experiment, the mini-ozone skid operator chose which ozone generator to use, what ozone concentration to target, and what water temperature to set.

##### 2.4.2.2 Ozone-bacteria reactions

A flow chart (Figure 2B) shows the experimental procedure used in Setup B. Cell suspension preparation was almost identical to that of Setup A, except that the cell suspension used in inoculating the reaction mixture contained 10^7^–10^8^ CFU/mL. The mini-ozone skid (Figure 1B) was used to produce ozone with concentration monitoring and control in real-time. Concentrations of ozone were verified using the Indigo method. Each run began by dispensing 1,000 mL of ozone-treated water in an autoclaved flask that has been pre-rinsed with aqueous ozone. The flask, containing a stir-bar, was placed on a stirring pad (model MS-H280-Pro; OniLAB, City of Industry, CA, USA) set to 400 rpm to allow thorough mixing without splashing. Immediately after dispensing the ozone-treated water, it was inoculated with 1 mL of the 10^7^–10^8^ CFU/mL cell suspension. The exact initial ozone level was recorded, then ozone concentrations were measured after 5–120 s, depending on the targeted ozone dose.

After each holding time, ozone concentration was measured with the Indigo method using 1 mL of the treated suspension. At the end of the reaction time, 100 mL of sterile 2.0% w/v sodium thiosulfate solution was added to the contents of the flask and the mixture was held for 30 s. The flask contents (1,100 mL total) were poured aseptically through a filter plate (Neogen). If the cell suspension was subjected to mild treatment conditions, the volume filtered was reduced to 100 mL. Filter plates were then incubated at 35°C for 48 h. For the untreated control, 1 mL of cell suspension was added to 1,000 mL of purified water and 100-mL of sodium thiosulfate was added. A 10-mL aliquot of the mixture was pipetted, and a 100-fold dilution was prepared from this aliquot. A portion (0.1 mL) of the dilution was plated on TSA in duplicate plates and incubated as described previously. After incubation of all plates, colonies were counted, and populations of survivors were estimated.

### 2.5 Ozone measurement

Several methods were used to measure ozone and comparisons were made between these methods to ensure consistency. The Indigo method (Bader and Hoigné, 1981) is the official ozone determination method, and it was used whenever possible, or when calibration of other methods was needed. An adaption of this method was used in Setup A and some Setup B experiments as follows. Indigo stock solution was prepared using a 100-mL volumetric flask. Potassium indigotrisulfonate (0.077 g) was mixed with 50 mL of deionized water, and then 0.1 mL of phosphoric acid was added to the mixture and stirred. Deionized water was then used to bring the total volume to 100 mL. This stock Indigo solution was then used to prepare the Indigo reagent as follows. A portion (10 mL) of the Indigo stock solution was dispensed in a 100-mL volumetric flask and mixed with 0.7 mL of phosphoric acid and 0.1 g of monobasic sodium phosphate; deionized water was used to bring the total volume to 100 mL. For determination of ozone in the 100-mL bacterial reaction volume (Setup A), 2 mL of the Indigo reagent (or 1 mL only if ozone concentration is expected to be less than 1 mg/L) were dispensed in each of two test tubes. Reactions samples (2 mL, each) were dispensed in the Indigo reagent tubes and the contents were mixed for 15 s. The OD_600nm_ of the mixtures was measured and absorbance difference from an ozone-free control (blank) was calculated. Then ozone concentration (mg/L) was calculated as follows:

$$O\,z\,o\,n\,e\,c\,o\,n\,c\,e\,n\,t\,r\,a\,t\,i\,o\,n\,\left(m\,g/L\right)=N^{*}\,D/\left(0.42^{*}\,b^{*}\,V\right),$$

where *N* = total volume (mL) (i.e., Indigo reagent volume + sample volume), *D* = difference in absorbance, *b* = path length (cm), and *V* = volume of sample (mL). For the 1,000-mL bacterial reaction volume (Setup B), ozone was determined by the Indigo method after the ozone treatment was completed. In this case, 1 mL of the reaction mixture was mixed with 1 mL or 0.5 mL of the Indigo reagent; the latter was used for ozone levels lower than 1 mg/L. The remainder of the procedure was completed as described earlier. Rosemount sensor was used in Setup B experiments where instantaneous ozone measurement and feedback control were required. The accuracy of the readings of the sensor was verified using a commercial ozone testing kit (K-7423 and K-7433; CHEMetrics, Midland, VA, USA). Testing kit results were also compared to the results of the Indigo colorimetric method to ensure ozone concentration measurement accuracy.

### 2.6 Determination of Ct

When ozone concentration varies during exposure of treated organisms, Ct value was calculated by integration of the measured concentration as function of time (e.g., Tizaoui et al., 2022). In the current study, a two-point trapezoidal integration method was used to determine the area under the concentration vs. time curve. The two points represented the starting and end points of ozone measurements. Both the starting concentration and the time factor were varied in different experiments although ozone concentration was more often varied, and treatment time was typically 30 s. The following equation was used to calculate the Ct value.

C⁢t=([O3]s-[O3]e)⁢t2*+([O3]e⁢t*),

where *Ct* is a concentration-time measurement, *[O_3_]_*s*_* is the ozone level at the start of reaction (mg/L) in the ozone-bacteria reaction mixture (Figure 2), *[O_3_]_*e*_* is the ozone level at the end of reaction (mg/L), and *t* is time (min).

In some statements of this work, “dose” was used in lieu of “Ct,” considering that both terms convey a similar meaning (EPA, 2023). Therefore, the terms “Ct” and “dose” will be used interchangeably to describe the strength of ozone treatment against a targeted microorganism.

### 2.7 Bacterial population count after compensation for dilution processes

The experiments performed included several critical and unavoidable processes that partially diluted the bacterial population in the reaction mixture. To determine the population of survivors accurately, these dilution processes were accounted for by correction procedures. The following is an example of the correction procedure that was implemented in some of the experiments. When 1,000-mL reaction volume was targeted (as described earlier in Setup B), 1 mL of culture was added before the reaction started, 1 mL of the ozone-cell reaction mixture was taken for ozone measurement after the reaction was completed, 100 mL of Na_2_S_2_O_3_ solution was added, and then a 100-mL sample was withdrawn for determination of the population of survivors using the filter-plate. In this example, the first step of the correction was to calculate the ratio of the initial volume containing the cell suspension to the final volume, i.e., the dilution ratio:

$$\begin{array}{l} D\,i\,l\,u\,t\,i\,o\,n\,r\,a\,t\,i\,o\\ =\frac{\begin{array}{c} 1000\,m\,L\,\left(o\,z\,o\,n\,e\,w\,a\,t\,e\,r\right)+1\,m\,L\,\left(c\,e\,l\,l\,s\,u\,s\,p\,e\,n\,s\,i\,o\,n\right)\\ -1\,m\,l\,\left(f\,o\,r\,o\,z\,o\,n\,e\,m\,e\,a\,s\,u\,r\,e\,m\,e\,n\,t\right) \end{array}}{\begin{array}{c} 1000\,m\,L\,\left(o\,z\,o\,n\,e\,w\,a\,t\,e\,r\right)+1\,m\,L\,\left(c\,e\,l\,l\,s\,u\,s\,p\,e\,n\,s\,i\,o\,n\right)\\ -1\,m\,l\,\left(f\,o\,r\,o\,z\,o\,n\,e\,m\,e\,a\,s\,u\,r\,e\,m\,e\,n\,t\right)\\ +100\,m\,L\,\left(N\,a\,t\,h\,i\,o\,s\,u\,l\,f\,a\,t\,e\right) \end{array}}\\ D\,i\,l\,u\,t\,i\,o\,n\,r\,a\,t\,i\,o=0.909 \end{array}$$

The ratio was used to calculate the volume of the original cell suspension that was treated.

$$\begin{array}{l} O\,r\,g\,i\,n\,a\,l\,v\,o\,l\,u\,m\,e\,u\,s\,e\,d=D\,i\,l\,u\,t\,i\,o\,n\,r\,a\,t\,i\,o^{*}\,s\,a\,m\,p\,l\,e\,d\,v\,o\,l\,u\,m\,e\,\left(m\,L\right)\\ \;=0.909^{*}\,100\,m\,L=90.9\,m\,L \end{array}$$

When calculating the final population of survivors, assuming that 203 colonies were counted on the incubated filter, the population count on the filter-plate associated to this original volume was determined as follows:

$$\begin{array}{l} C\,F\,U/m\,L=\,\frac{c\,o\,u\,n\,t}{o\,r\,g\,i\,n\,a\,l\,v\,o\,l\,u\,m\,e\,u\,s\,e\,d\,\left(m\,l\right)}=203\,C\,F\,U/90.9\\ \;m\,L=\,2.23\,C\,F\,U/m\,L \end{array}$$

### 2.8 Data analysis for model development

The variables tested in this study were ozone concentration, treatment time, and log reduction in the populations of treated bacterial strains. Each experiment was run at a desired ozone Ct value; hence, ozone concentration and treatment time were predetermined. Although treatment times were controlled reasonably accurately, measured ozone often deviated slightly from the intended concentration. Consequently, runs intended to produce the same Ct value were treated as independent trials rather the replicates.

Simple linear regression (SLR) was used to characterize the relationship between Ct and log reduction in cell populations. However, using SLR to determine the lower bounds of these relationships could not be directly determined due to data heteroscedasticity, i.e., unequal variance in log reductions as the Ct value changes (Supplementary Figure 1). A key assumption for SLR validity is data homoscedasticity (Richter and Piepho, 2019), where dispersion of data points about the regression trend line is to be equal over the range of measured values (i.e., constant variance of the residuals). Due to this, homoscedasticity is required for determination of efficient ordinary least squares (OLS) estimators (i.e., least variance regression coefficients), appropriate assessment of statistical significance, as well as accurate confidence intervals. However, these assumptions were not met in the dose-response data collected in the current study. Additionally, the interest in this study was to determine the lower bound of this heteroscedastic linear regression relationship, but such a method is believed to be unavailable in published literature. Hence, an approach has been developed to address this problem, which is detailed in the Results section.

Data analysis was performed via Minitab 17 Statistical Software (2010), Microsoft Excel 2019, and Real Statistics Resource Pack Software (Release 7.6). Statistical significance was calculated at 95% confidence unless noted otherwise.

## 3 Results

When the investigated bacteria (Table 1) were treated with different ozone concentrations and treatment times (i.e., different Ct values), a decline in the populations’ log_10_ CFU/mL (i.e., Δ log_10_ CFU/mL, or simply “log-reduction”) increased with increasing the Ct value. The trend seen in this dose-response relationship varies with the tested species and stain. Although linear dose-response relationships were obvious in many of the data sets, data scatter around the regression line was often uneven (Supplementary Figure 1); hence, it was prudent to use a meaningful statistical approach to analyze these data before conclusions can be made. The primary interest herein is not in the statistical significance of the dose-response linear regression relationship itself, but rather in characterizing the lower bound associated with this relationship. The lower bounds of these scattered dose-response data would represent useful and practical dose-response relationships; each data point on this lower bound represents an ozone Ct value sufficient to cause the corresponding log-reduction value, at least. The following sections cover how statistical models were developed and used to analyze the data collected in the current study.

### 3.1 Model development

**FIGURE 3:**
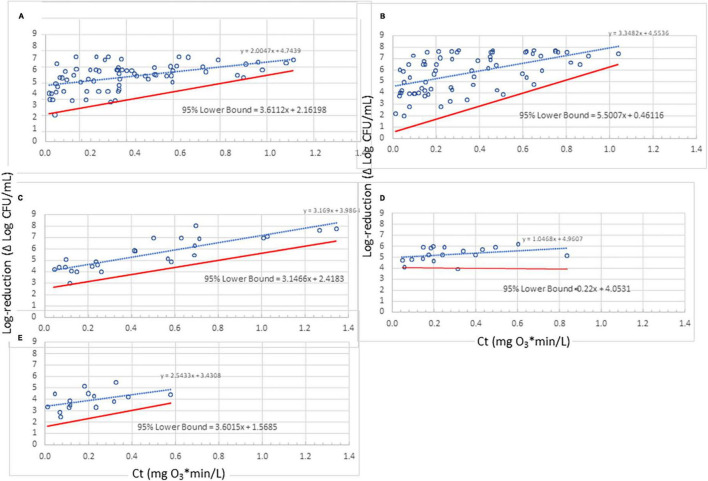
Lower bounds for ozone dose (mg O_3_*min/L)-response (log-reduction) relationship for 10 strains representing five bacterial species. **(A)** *Enterococcus faecium*; **(B)** *Salmonella enterica* serovars Livingstone, Tennessee, and Typhimurium; **(C)** *Pseudomonas aeruginosa*; **(D)** *Listeria monocytogenes* strains California, Scott A, and V7; **(E)** *Escherichia coli* serotypes K12 and O157:H7. Despite their small data sets, the lower bounds for panels **(C,E)** seem reasonable whereas that for panel **(D)** does not. Use of a lower bound derived with standard methods (assuming homogeneity) may be more appropriate in some cases like panel **(D)**; therefore, judgment needs to be used with very small data sets in the development of a lower bound.

When many of the data gathered in this study (Ct vs. log-reduction) were presented graphically, the dose-response plots exhibited linear relationships but with “funnel-shaped” spread about these best-fit lines, i.e., heteroscedastic trends (Figure 3). This heteroscedastic trend is in part related to the limit of detection (LOD) of the enumeration method, which was ∼0.01 CFU/mL (Setup A of Figure 2) or ∼0.001 CFU/mL (Setup B of Figure 2). When the Ct values increased, the population in treated water decreased until the method’s LOD (∼0.01 or 0.001 CFU/mL) was reached. Considering that water was inoculated at 10^4^ to 10^5^ CFU/mL, the maximum detectable reduction in treated cell population was in the range of 6–8 log. Therefore, the data’s “funnel-shape” was due in part to LOD of the enumeration method as shown in Supplementary Figure 2. These inherent circumstances resulted in data heteroscedasticity. The data heteroscedastic trend made it inappropriate to use conventional statistical analysis (e.g., SLR), which assumes homoscedasticity to draw conclusive outcomes. Additionally, practical application of these results necessitates determining the lower bound of the dose-response relationships; such a lower bound would be industrially valuable considering that it represents the ozone dose needed to address the worst-case for microbial contamination of water.

#### 3.1.1 Characterizing the lower bound of a heteroscedastic simple linear regression relationship

**FIGURE 4:**
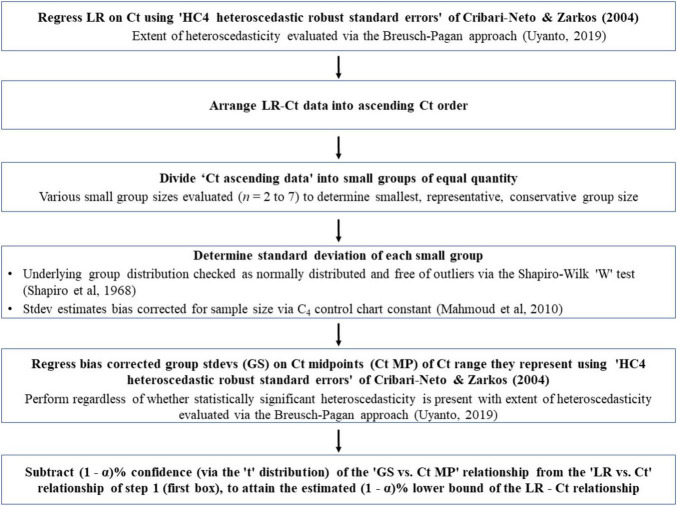
Flowchart of a novel method to determine the lower bounds of heteroscedastic log-reduction (LR) vs. ozone dose (mg*min/L; Ct) relationships. Main steps are shown in bold-face and sub-step methodologies used to verify key assumptions are in regular text.

With heteroscedasticity, OLS estimators are still linear and unbiased, but can provide misleading results regarding the statistical significance of the relationship. Potentially misleading t and F tests are caused by inappropriate standard errors of the OLS estimates (Wilcox and Keselman, 2004). To overcome the potential adverse effects of inappropriate standard errors of OLS estimates, heteroscedastic ‘robust standard errors’ are to be utilized (Cribari-Neto and Zarkos, 2004). Following what Cribari-Neto and Zarkos concluded, we have used the ‘HC4 heteroscedastic robust standard errors.’ Built upon this, a novel method has been developed to determine the lower bound of a heteroscedastic relationship. The method has six steps as shown in Figure 4. Sub-step methodologies, used to verify key assumptions (discussed momentarily) are also shown unemboldened in Figure 4. The developed method has the following core elements:

(a) Arrange the observed ‘Ct vs. log-reduction’ pairs in an ascending Ct order.
(b) Divide this sorted list into small sequential groups of equal quantity (providing equal precision of grouping standard deviation estimates, as mentioned in the next step), thereby creating a collection of small groups, each representing log-reduction results for the small range of Ct they came from.
(c) Determine the standard deviation of each group.
(d) Regress the resultant standard deviation against the mid-points of the Ct ranges they represent.
(e) For determination of the lower bound, subtract the resultant linear model regression equation (multiplied by the appropriate number of standard deviations for the desired confidence, i.e., ∼ 2.26 standard deviation for a 95% lower bound based on 10 groups; via the “t” distribution) from the linear model equation for log-reduction as a function of Ct.

To be statistically sound, the above approach must meet the following conditions: (i) the small groups do not suffer from significant outliers in terms of log-reduction values; (ii) the underlying distribution of log reductions within these small groups can be reasonably assumed as normally distributed, and (iii) an unbiased estimate of each grouping’s standard deviation of log-reduction can be appropriately made. To test for outliers and evidence of non-normality in very small samples, researchers found that the Shapiro–Wilk “W” test is the most appropriate choice (Shapiro et al., 1968; Pearson et al., 1977; Zylstra, 2007; Le Boedec, 2016). Shapiro et al. (1968) evaluated the W method for samples as small as *n* = 10, hence, the W test has been employed here. Meeting the third condition, i.e., obtaining unbiased standard deviation estimates for the very small sample sizes encountered, required employing knowledge from the statistical quality control (SQC) discipline, which utilizes an approach for unbiased estimation of standard deviation of tiny groupings, as low as *n* = 2. Mahmoud et al. (2010) stated that a sample’s standard deviation derived the classical way (via sums of squared deviations from the mean) and then bias-adjusted (i.e., inflated) via use of the control chart constant “c_4_” is more efficient than the use of ‘range-based methods’ for tiny sample size cases. Hence, the sample’s standard deviation derived the classical way has been used here, then bias-corrected using the control chart constant “c_4_” obtained from the following equation:

$${\hat{\sigma }}_{L\,R}=S_{L\,R}/c_{4}$$

where, ${\hat{\sigma }}_{L\,R}$ is the estimated grouping’s standard deviation, *S*_*LR*_ is the sample standard deviation (derived via standard sums of squared deviations from the mean approach), and c_4_ is control chart constant (Supplementary Table 1).

#### 3.1.2 Lack of normality and presence of outlier’s check

Ten strains representing five bacterial species were evaluated. The number of observations for each species varied (Table 2). As outlined earlier, the W test was used to indicate evidence of outliers and/or lack of normality, both being potential conditions existing with these datasets. Table 2 shows *p*-values for a sampling of W tests of various small group sizes (all *n* = 10 or slightly larger) for the species tested in this study. Based on this sampling of small group size outcomes (Table 2), it is the authors’ opinion that normality along with a lack of significant outliers are reasonable assumptions regarding the underlying circumstances for the small groupings of these datasets.

**TABLE 2:** Tests of normality (and for outliers) via the Shapiro-Wilk test for various tested species using group sizes of 10 or slightly larger.

		*p*-value per Shapiro-Wilk test for group sizes of *n* ≥ 10* (groups created by grouping ascending values of *n* ≥ 10, each)										
Species	Total observation count	1^st^ Group of 10	2^nd^ Group of 10	3^rd^ Group of 10	4^th^ Group of 10	5^th^ Group of 10	6^th^ Group of 10	1^st^ Group of 11	1^st^ Group of 12	1^st^ Group of 13	1^st^ Group of 15	1^st^ Group of 18
*Enterococcus faecium*	68	0.93	0.84	0.48	0.20	0.27	0.17					
*Escherichia coli*	15										0.93	
*Listeria monocytogenes*	18											0.22
*Pseudomonas aeruginosa*	25								0.64	0.39		
*Salmonella serovars*	71	0.84	0.22	0.08	0.20	0.06	0.04	0.47				

*Groups of 10 are considered the smallest group size that can reasonably be assessed with the W test; therefore, for *E. faecium* for instance, with its 68 observations, has 6 groups of 10 and a 7^th^ group with 8 (not evaluated).

#### 3.1.3 Group size check

As outlined in Figure 4, the third step involved dividing “Ct ascending data” into small groups of equal quantity. The larger the number of groups, the better the comparison of the lower bounds among tested species. However, there is a tradeoff; more groups mean a smaller number of observations in each group, and this could have adverse effects on the analysis. To determine the suitable group size, lower bounds based on *n* = 2–7 group sizes were evaluated for two bacterial datasets of interest, *E. faecium* (Dataset 1) and all other species (Dataset 2). The results of these analyses are shown in Supplementary Figure 3. As can be seen, the estimated 95% lower bounds differ due to group sample size, with *n* = 2 and *n* = 3 bounds appearing particularly different as compared to bounds based on higher sample sizes. Additionally, *n* = 4 to *n* = 7 bounds appear fairly comparable to one another for the low Ct rates. Therefore, *n* = 4 grouping has been chosen for comparing the lower bounds of Dataset 1 and Dataset 2; this allowed for satisfactory discerning power (i.e., power that a difference between lower bounds exists) due to a higher number of groups, while being comparable to bounds derived from the larger sample sizes. Determining optimal group sample size with this methodology requires additional investigation with datasets other than those covered in current study.

#### 3.1.4 Heteroscedasticity check

As mentioned in a previous section, the extent of heteroscedasticity (for Ct vs. log-reduction) was evaluated via the Breusch-Pagan approach (Uyanto, 2019). This approach has been applied for Dataset 1, Dataset 2, as well as all data observed in the study (Dataset 3), as shown in Supplementary Figure 4. Although all three datasets appear heteroscedastic, Dataset 2, with *p*-value = 0.086, technically does not meet the 95% confidence that evidence of heteroscedasticity exists; this result is due to scarcity of observations in the higher Ct ranges, i.e., Ct > 0.90. Despite a lack of statistical evidence, this level of heteroscedasticity, however, may still adversely affect the t- and F-tests due to inappropriate standard errors, as discussed previously. Consequently, the use of ‘HC4 robust standard errors’ has been used and is advised as a conservative approach (unless one clearly has a homoscedastistic circumstance, i.e., consistent dispersion of results across the range of Ct values tested).

#### 3.1.5 Lower bound determinations

**FIGURE 5:**
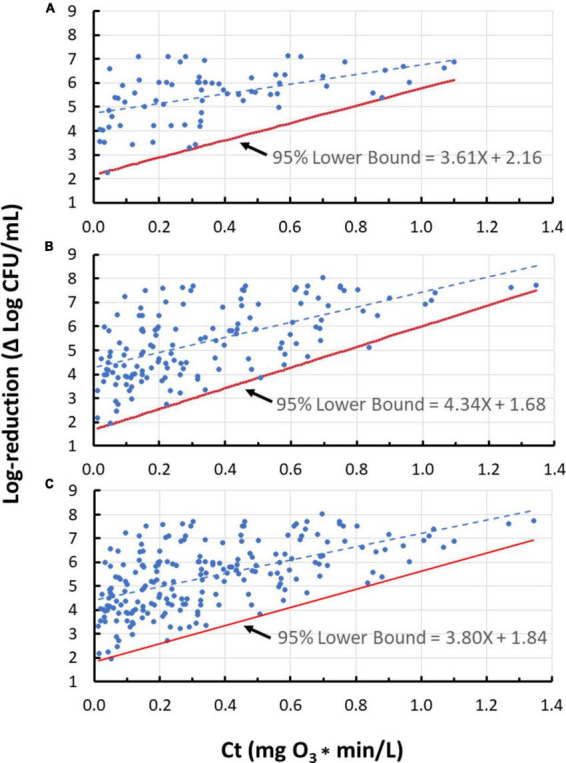
The 95% lower bound of ozone dose (Ct value) vs. log-reduction for *Enterococcus faecium* only **(A)**, all tested species excluding *E. faecium* **(B)**, and all tested species **(C)**, using *n* = 4 standard deviation group sizes.

Lower bounds have been estimated for all three datasets employing the method outlined previously (Figure 4) and the results are shown in Figure 5. It is important to note that *n* = 4 standard deviation group sizes have been used as discussed in Section “3.1.3 Group size check.” Additionally, to determine 95% confidence of the lower bound, the “t” distribution was used, thereby taking into account the number of standard deviations (i.e., standard deviations groups) involved.

#### 3.1.6 Comparing the lower bounds for *E. faecium* (Dataset 1) and all other species (Dataset 2)

**FIGURE 6:**
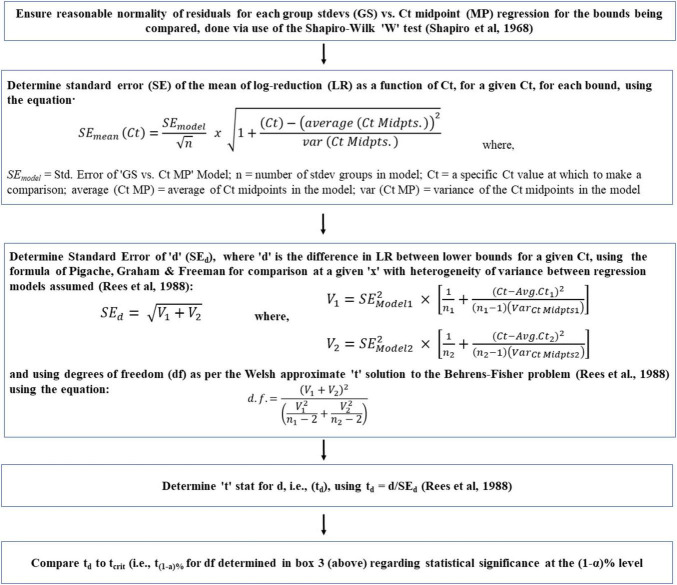
Approach used to compare lower bounds of various species or dataset at an ozone dose (Ct, mg*min/L) of interest.

**FIGURE 7:**
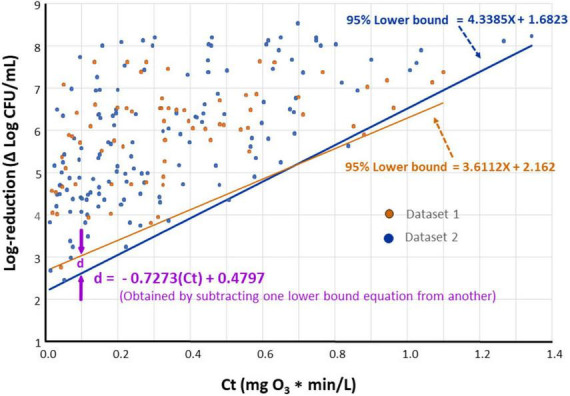
The 95% lower bounds of ozone dose (Ct value) vs. log-reduction of *Enterococcus faecium* (Dataset 1, orange) and all species excluding *E. faecium* (Dataset 2, blue) when *n* = 4 standard deviation group sizes are used.

It is of interest to compare lower bounds of dose-response relationships for various datasets. For example, if the lower bounds of datasets 1 and 2 are indistinguishable, it may be concluded that *E. faecium* can serve as a surrogate for the pathogens tested under dataset 2. A statistical approach was developed (Figure 6) to compare the lower bounds of different species or datasets at specified Ct values. Utilizing this methodology, a comparison was made between lower bounds for Dataset 1 and Dataset 2 (Figure 7). In this comparison, “d” represents the difference between bounds at a given Ct. As a first step in this comparison (Figure 6), normality of residuals for each group standard deviation vs. Ct mid-point regression was checked and found sufficiently normal via the “W” test. Subsequent steps of the approach, detailed in Figure 6, have been carried out to determine if there is statistical evidence to suggest a difference in the lower bounds of these two datasets. The results of these steps are shown in Table 3.

**TABLE 3:** Statistical comparison of lower bounds (d) for various Ct values of datasets 1 and 2, using the methods described in Figure 6.

Ct^1^	D^2^	SE_mean_ (Ct)^3^ (Dataset 1)	SE_mean_ (Ct) (Dataset 2)	V_Dataset1_^4^	V_Dataset2_	SE_d_^5^ [√(V_Dataset1_ + V_Dataset2_)]	Var_d_^6^	t^7^ (d/SE_d_)	d.f.^8^	t_crit_^9^ (a = 0.05, d.f. as shown)	Confidence of a difference
0.025	0.46	0.188	0.140	0.036	0.020	0.237	0.056	1.95	36	2.028	94.0%
0.050	0.44	0.180	0.133	0.033	0.018	0.224	0.050	1.98	34	2.032	94.4%
0.075	0.43	0.172	0.127	0.031	0.016	0.214	0.046	1.99	33	2.035	94.5%
0.100	0.41	0.165	0.122	0.028	0.015	0.205	0.042	1.99	33	2.035	94.5%
0.200	0.33	0.139	0.103	0.020	0.011	0.173	0.030	1.93	33	2.035	93.7%
0.300	0.26	0.123	0.093	0.015	0.009	0.155	0.024	1.69	34	2.032	90.0%
0.400	0.19	0.120	0.095	0.015	0.009	0.153	0.024	1.23	35	2.030	77.3%

^1^Ct: concentration x time (i.e., dose);

^2^d: difference in log reduction between lower bounds for a given Ct;

^3^SE_mean_: Standard error of the mean of log-reduction, as a function of Ct;

^4^V: variance of Ct for a given dataset;

^5^SE_d_: standard error of d;

^6^Var_d_ – variance of d;

^7^t : t statistic;

^8^d.f.: degrees of freedom;

^9^t_crit_ – t statistic with α error = 0.05.

#### 3.1.7 The lower bounds for dose-response relationship of the five species tested

Using the methodology described previously (Figure 4), lower 95%-confidence bound results have been obtained for the five species tested in the current study (Figure 3). To determine 95% confidence, the “t” distribution was used, thereby taking into account the number of standard deviations involved. Sample group sizes were *n* = 4 for *E. faecium* and *Salmonella* serovars, and *n* = 2 for the other species (due to their small number of observations). Three of these species (*P. aeruginosa*, *L. monocytogenes*, and *E. coli*) do not exhibit substantial heteroscedasticity and could be treated as homoscedastic but have been included here for illustration with this methodology; the non-heteroscedasticity for the three bacteria could be due to lack of sufficient data points collected or due to inherent traits in the tested species.

### 3.2 Ozone antimicrobial efficacy in purified water, determined using the lower bound of dose-response relationship

The pooled dose-response data, collected for all the tested bacteria, could be useful in ensuring the safety of purified water regardless of the cause of contamination. Figure 5C depicts dose-response data compiled for all strains of the five tested species. The 95%-confidence lower bound of this dose-response relationship is represented by the following equation:

L⁢o⁢g⁢p⁢o⁢p⁢u⁢l⁢a⁢t⁢i⁢o⁢n⁢r⁢e⁢d⁢u⁢c⁢t⁢i⁢o⁢n=3.80⁢C⁢t+1.84

Using this relationship, it will be possible to determine the ozone Ct value sufficient to achieve any desired population reduction within the tested Ct range, with approximately 95% confidence (Table 4). For example, to accomplish 5-log reduction in any of the members of the set of bacteria analyzed in this study, a Ct value of 0.832 mg O_3_*min/L is needed. Using this information, one can determine other combinations of ozone concentrations and treatment times, having the same ozone Ct value that can accomplish 5-log reduction. For example, 0.416-mg/L ozone treatment of purified water for 2 min could also result in 5-log reduction, or more, in any of the tested species. It may be worth noting that when experiments were performed side-by-side to test a given Ct, using different ozone concentrations and treatment times, similar log-reductions in tested populations were achieved (data not shown). In a previous study, ozone Ct of ∼0.4 at 20°C was sufficient to inactivate 3 log of viruses, but a Ct of ∼0.72 at 20°C was needed to inactivate 3 log of the cysts of *Giardia* spp. (EPA, 1999).

**TABLE 4:** Estimated reduction in bacterial populations (Δ log_10_ CFU/mL) based on the lower bound model deduced from the data collected in the current study using pathogenic and non-pathogenic bacteria.

Population reduction (Δ log_10_ CFU/mL)	Required ozone dose (Ct value) at 21°C
2	0.042
3	0.305
4	0.568
5	0.832
6	1.095

It should be cautioned that the lower bound model is valid only within the Ct range tested in the current study. Although applying the smallest Ct value tested (i.e., 0.01 mg O_3_*min/L) resulted in a considerable reduction in the tested bacteria, the inactivation kinetics at smaller Ct values is not known but very likely to deviate from the dose-response relationship described by the above equation. Data from a previous study (Kim and Yousef, 2000) implied a two-stage dose-response relationship; rapid increase in log reduction was observed at the short first stage, and slower increase at the second stage. The starting point of the first stage is expected to be zero inactivation at zero Ct value, which corresponds to the untreated control. Therefore, it is proposed, conservatively, that the above equation is valid through a Ct range of 0.1–1.1 mgO_3_*min/L; this range corresponds to 2.22–6.02 log-reductions in bacterial populations in water.

### 3.3 Suitability of *Enterococcus faecium* as a surrogate for the tested pathogens

Based on results shown in Figure 7, negligible differences were found in the lower bounds of dose-response plots for *E. faecium* ATCC 8459 (Dataset 1) compared to that for all tested species excluding *E. faecium* (Dataset 2). This finding provides sufficient confidence to suggest *E. faecium* as a surrogate for the bacterial pathogens tested in the current study during treatment of purified water with ozone. In previous studies, this *E. faecium* strain was also considered a suitable surrogate that can be used in lieu of foodborne pathogens in process validation for different products (Kopit et al., 2014; Smith et al., 2014).

### 3.4 Resistance of treatment survivor to ozone

Preliminary experiments were completed to determine if the bacterial population that survives ozone treatment gained resistance to subsequent ozone treatment. Colonies of *E. faecium* recovered from ozone treatment experiments were cultured and their resistance to ozone was compared to that of an untreated culture. Cell suspensions from both culture sources were found to exhibit similar ozone sensitivities (data not included). Additional experiments should be completed in the future to confirm this observation.

## 4 Discussion

### 4.1 Choice of experimental design

High inoculation levels (e.g., 10^6^–10^9^ CFU/mL) in various matrices are commonly used in microbial inactivation kinetic studies, particularly when physical lethal factors such as heat are used. When such high inocula are used to study ozone inactivation kinetics in a batch aqueous system, microbial populations decrease quickly (e.g., in 30 s or less) to a level that depends on the applied ozone dose, then the populations change less rapidly during the remainder of the treatment (Kim and Yousef, 2000). Many previous findings suggest that while ozone exerts its antimicrobial action, it is consumed by the treated microorganisms, which constitute an organic load that has ozone demand. To emphasize ozone’s antimicrobial potential and minimize its consumption by the organic load, low microbial inoculation levels (10^3^–10^4^ CFU/mL) were used in the current study. Despite the low microbial levels tested, these are still higher than the expected water bioburden at the time of ozonation. Additionally, membrane filtration of large volumes (100–1,000 mL) of reaction mixtures was employed to decrease the minimum detection limit of the treated populations. Despite the low inoculation levels used, the procedure just described allowed measuring 6–8-log reductions in treated microbial populations. This approach should still represent a worst-case scenario in the manufacturing of purified bottled water. To address the microbiological quality of bottled water, the FDA regulations stipulate that coliform count shall not exceed 1/100 mL of that water (Code of Federal Regulations, 2023c).

### 4.2 Usefulness of modeling the lower boundaries of the dose-response relationships

When inoculated purified water was treated with ozone, log reduction in the microbial populations (i.e., response) increased linearly as a function of the applied ozone dose (i.e., Ct) despite the large variations in responses at a given dose, particularly at the small Ct values (e.g., Figure 7). Most of these variations were predictably due to the five species and ten strains we tested. However, minor but unavoidable discrepancies in culture preparation procedure, inoculum size, type of purified water, room temperature, method of capturing survivors for enumeration, and other factors may have contributed to the changes in responses seen at the same dose. For example, in a previous study (Yesil et al., 2017), it was found that log-reduction results were strongly dependent on the inoculum size of the treated microorganism. It was presumed that cells killed in the high-inoculum reaction mixture released greater ozone-demanding cytoplasmic material, compared with the cells in the low-inoculum reaction mixture.

Despite the importance of the dose-response linearity, the lower bound of this relationship is the most relevant to the safety of purified water. A lower bound for a dose-response relationship represents ozone Ct needed to address the most resistant sub-population of the tested pathogen. Considering the large number of independent trials, which were completed in more than 24 months of testing, the resulting dataset is expected to represent various inadvertent physiological or experimental variations in the tested bacteria. Therefore, the lower bound seen in the current study offers a conservative estimate of the worst-case scenario for purified water decontamination by ozone.

Modeling the lower bound for the heteroscedastic data required an unconventional statistical approach. This modeling was successfully completed with a novel statistical approach the authors developed, and the lower bounds were determined with acceptable confidence. To the knowledge of the authors, this statistical approach has not been reported before, and it represents a viable solution to derive insights from datasets showing heteroscedastic trends. Previously, lower bounds were implemented when treatments sufficient to prevent the growth of *Clostridium botulinum* and toxin production in cheese products were investigated (Tanaka et al., 1986; Glass et al., 2017). Despite the significance of these two studies, the lower bounds needed to ensure the safety of cheese products were determined by methods that were not detailed in these publications. Developing and using a model representing a lower bound for a dose-response relationship would be useful for determining ozone adequacy in eliminating the pathogen of interest. Considering the relatively large number of strains and species tested in the current study, the lower bound for the pooled dose-response data would be useful in determining the minimum ozone dose needed to assure the safety of treated water against the pathogens included in the study.

### 4.3 Industrial applications

The water industry can benefit from the models used herein to determine the lower boundaries of the Ct vs. log-reduction relationships, i.e., the dose-response relationships. Using this relationship for all tested bacteria in this study, it was concluded that ozone Ct of 0.832 (measured at 21°C) was sufficient to cause 5-log reduction in the studied set of bacterial pathogens, which are potentially of concern in purified water. Processors may apply this relationship to derive the required Ct that achieves the desired log reduction; thus, it can be used to justify the microbiological safety of purified water and adequacy of processes involved in making this water. Processors also can determine Ct values sufficient to accomplish safety goals other than the 5-log reduction. In purified water treatment, ozone is usually delivered through the use of ozone contact tanks. The dose-response relationship, concluded in the current study, could be used to derive different ozone concentration and contact tank time combinations. This relationship can also be used to evaluate process deviations where the required ozone Ct target is not met. It should be re-emphasized that the concluded dose-response relationship only applies to bacteria tested in the current study, and within the Ct range tested. Despite the importance of the bacteria tested in the current study, protozoa and viruses are pathogens of interest in some water treatment facilities.

One of the objectives of the current study was to determine if *E. faecium* ATCC 8459 (NRRL B-2354) would qualify as a surrogate for waterborne pathogens in ozone treatments; such a surrogate would facilitate studies aiming at decontaminating various waters by ozone. This *E. faecium* strain is a nonpathogenic bacterium that does not carry antimicrobial resistance genes (Kopit et al., 2014), and hence, is suitable for processing facility testing. The strain has been widely used in process validations of low moisture foods (Anderson and Lucore, 2012; Rachon et al., 2016; Dhowlaghar and Zhu, 2022), peracetic acid washing of apple (Zhu et al., 2021), peanut roasting (Smith et al., 2014), and others. Results of this study showed that the lower bounds of the dose-response relationship for the *E. faecium* strain and that for the other tested bacteria did not differ significantly (Figure 7). The figure, however, shows that the slight difference between these two boundaries, as represented by “d” value, varied depending on the Ct tested; the maximum d was < 0.5 log. There are three proposed options that can be implemented to correlate the surrogate’s dose-response data to those for pathogenic bacteria. The simplest option is to apply no correction factor considering that the lower bounds of the surrogate and the pathogenic bacteria are not significantly different (Figure 7). In another option, a correction factor of “d” is applied as expressed in the following equation (Figure 7):

$$d=-0.07273^{*}\,C\,t+0.4797$$

The third option would involve subtracting 0.5 log from surrogate’s log reduction values when the applied ozone Ct is less than ∼0.6.

## 5 Conclusion

The deduced conservative dose-response relationship for the tested pathogenic and non-pathogenic bacteria allows the bottled water industry to establish minimum ozone treatments sufficient to assure the safety of purified water. A strain of *E. faecium* was found to be a viable surrogate for potential pathogens of concern in purified water. The strain would be beneficial in validating ozone efficacy in bottled water processing facilities. Future research is needed to complement the current findings; such research could address the effects of water pH, temperature, and total dissolved solids on ozone treatment efficacy.

## Data availability statement

The raw data supporting the conclusions of this article will be made available by the authors, without undue reservation.

## Author contributions

YL: Conceptualization, Data curation, Investigation, Methodology, Visualization, Writing—original draft. DK: Data curation, Investigation, Methodology, Formal analysis, Writing—review and editing. ZH: Data curation, Investigation, Writing—review and editing. ZL: Writing—review and editing, Conceptualization, Funding acquisition, Supervision. DS: Writing—review and editing, Investigation, Methodology. RF: Methodology, Writing—review and editing, Data curation, Writing—original draft. AY: Writing—review and editing, Conceptualization, Funding acquisition, Project administration, Resources, Supervision.
