# Supplementary material for: Inactivation kinetics of selected pathogenic and non-pathogenic bacteria by aqueous ozone to validate minimum usage in purified water
Source: Front Microbiol. 2024 Jan 15;14:1258381. doi: 10.3389/fmicb.2023.1258381 (PMC10829095; doi:10.3389/fmicb.2023.1258381)
Supplement: Supplementary file 1 [file Presentation_1.pdf]

Figure S1

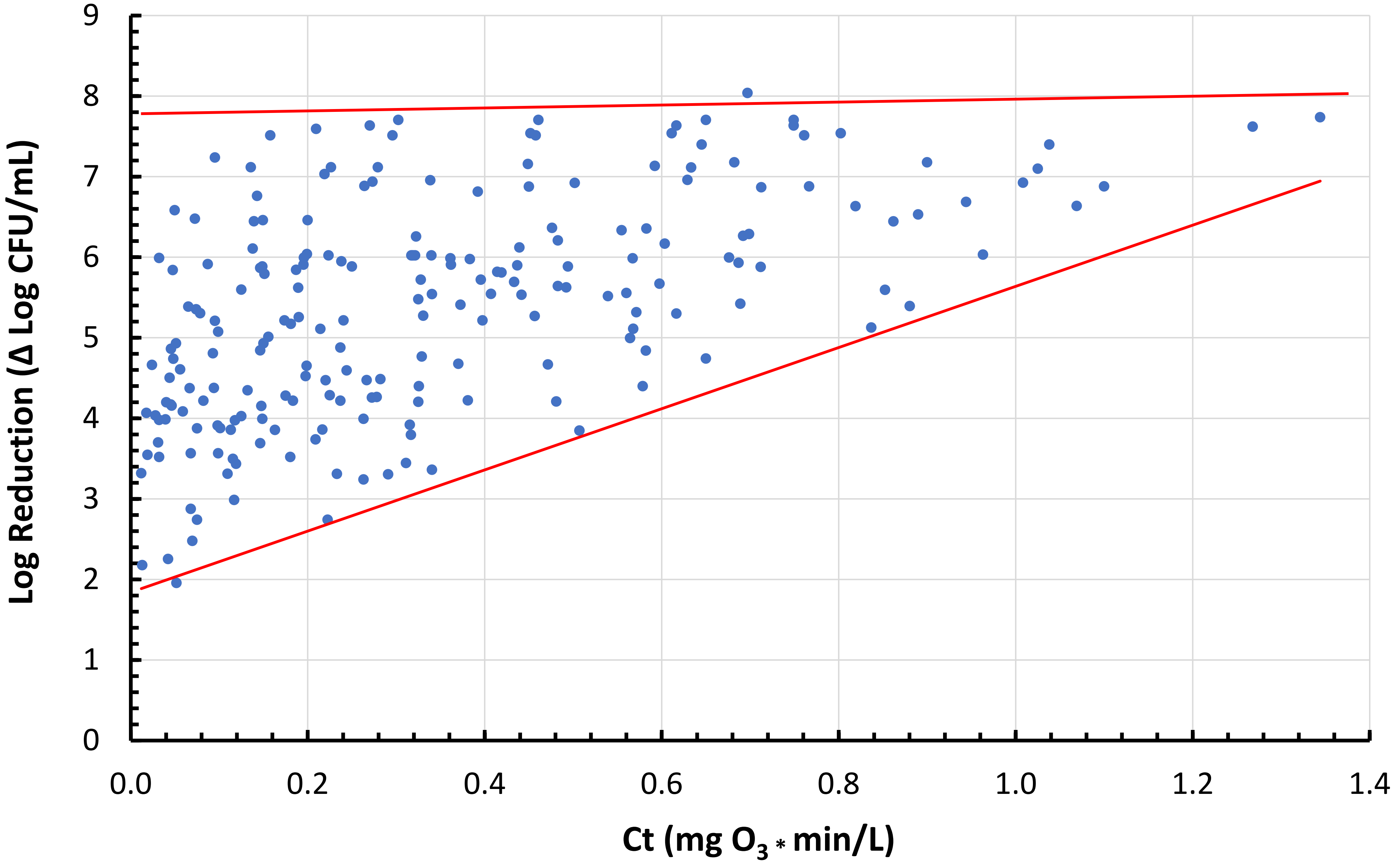

Figure S1. Dose-response plot for pooled results collected in this study showing data heteroscedasticity.

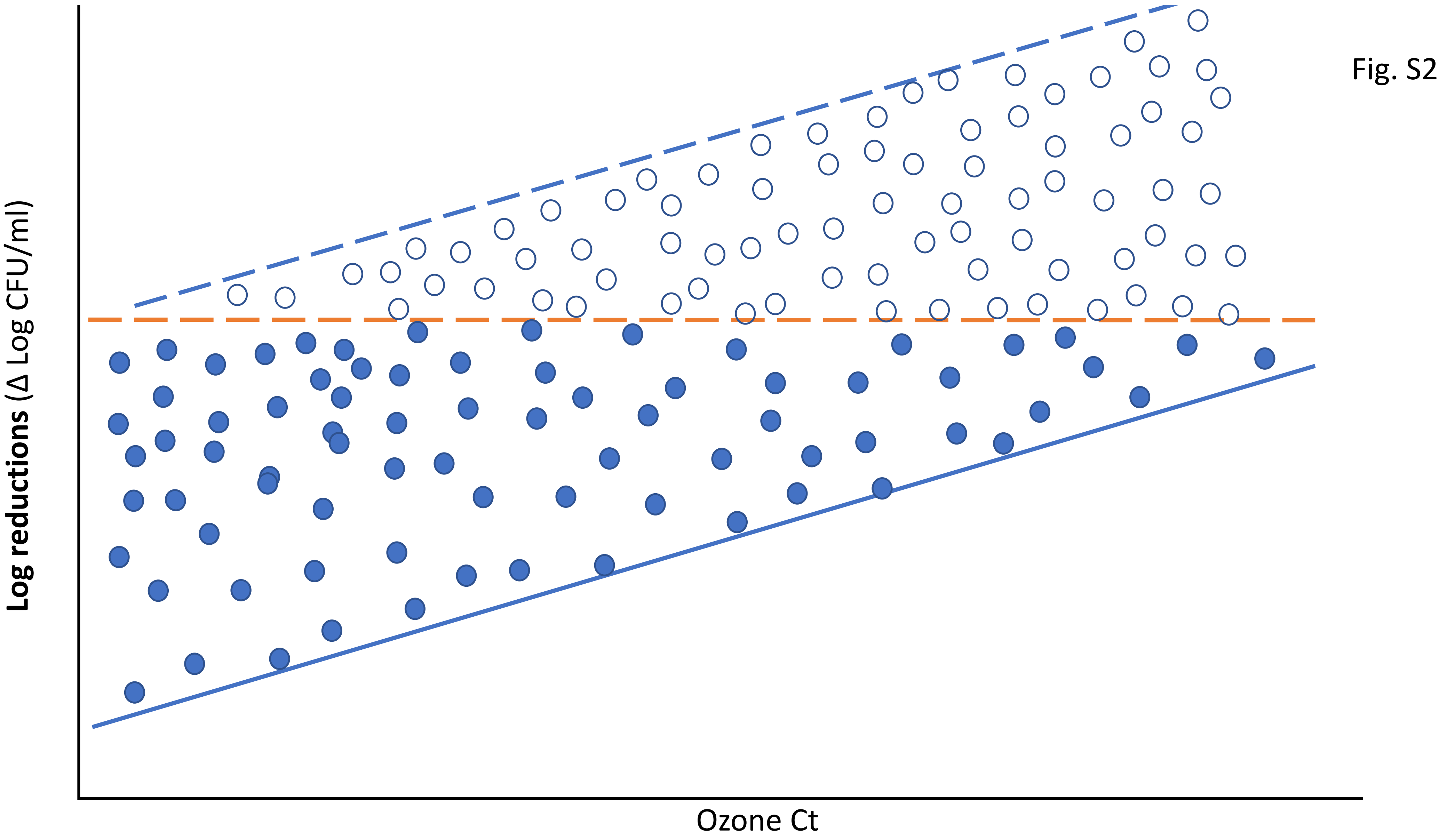

Figure S2. Hypothetical illustration showing how data heteroscedasticity likely formed in the current work. Solid circles represent a data set, while hollow circles represent hypothetical data that would likely exist if the enumeration's limit of detection (dashed red line) was located at higher log-reduction values.

Figure S3

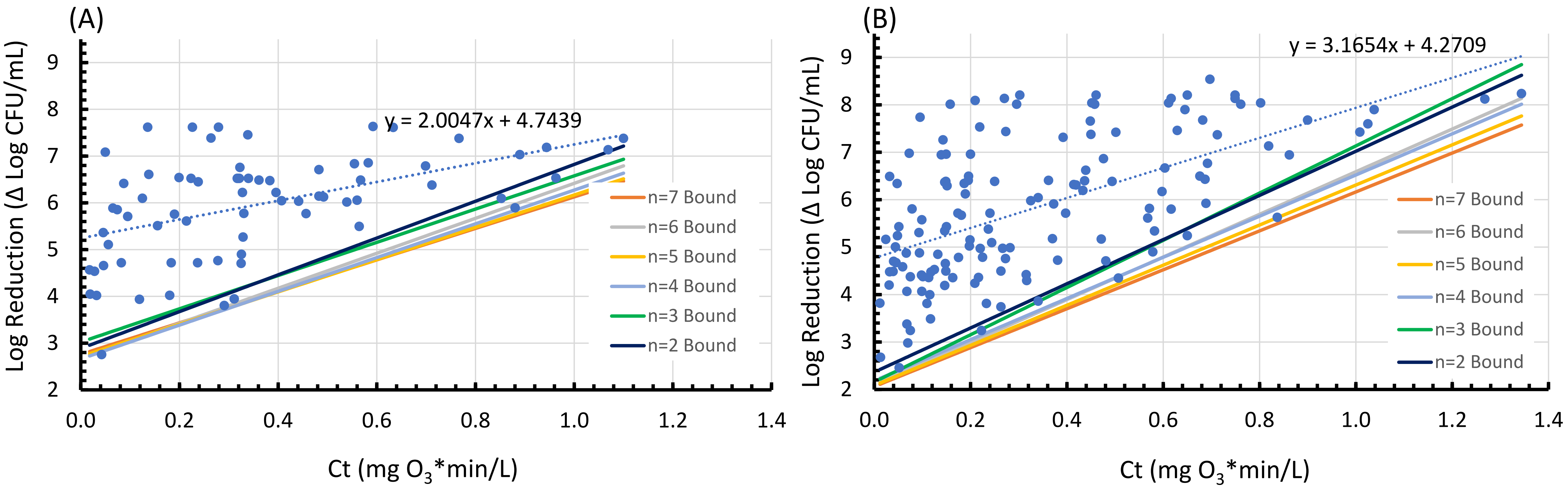

Figure S3. Comparison of estimated 95% lower bounds by group size for two bacterial datasets; *Enterococcus faecium* (Dataset 1, panel A) and all other tested species (Dataset 2, panel B).

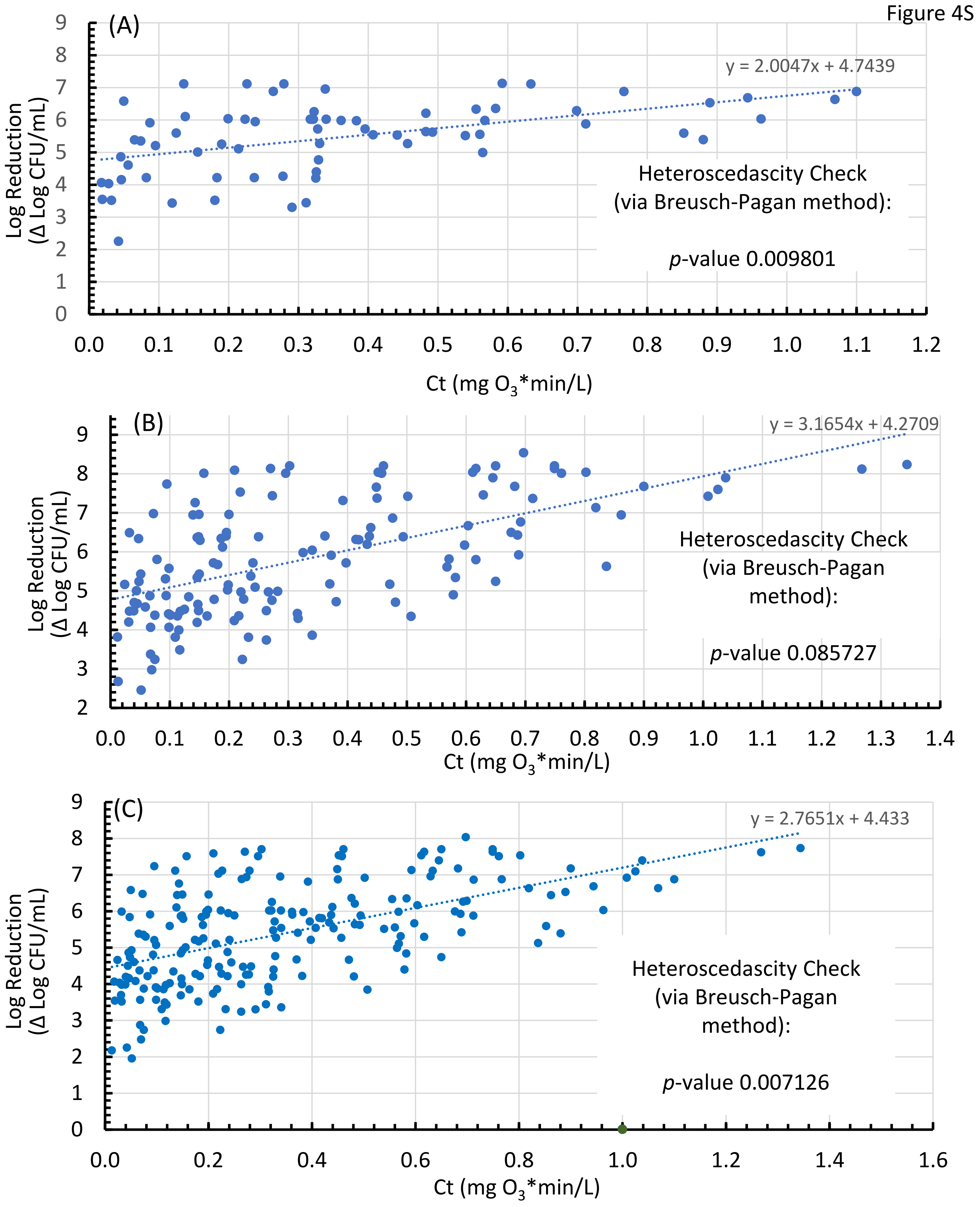

Figure S4. Heteroscedasticity assessments via the Breusch-Pagan approach. Data represent ozone dose (Ct-value) vs. log-reduction relationship for *Enterococcus faecium* only (Dataset 1, panel A), and all tested species excluding *E, faecium* (Dataset 2, panel B), and all tested species (Dataset 3, panel C).
