# Supplementary material for: Inactivation kinetics of selected pathogenic and non-pathogenic bacteria by aqueous ozone to validate minimum usage in purified water
Source: Front Microbiol. 2024 Jan 15;14:1258381. doi: 10.3389/fmicb.2023.1258381 (PMC10829095; doi:10.3389/fmicb.2023.1258381)
Supplement: Supplementary file 2 [file Table_1.DOCX]

Table S1. Control chart constant, c_4_, for sample sizes (n) from 2 to 20 (Mahmoud et al, 2010).

| **n** | **c_4_** | **n** | **c_4_** | **n** | **c_4_** |
| --- | --- | --- | --- | --- | --- |
| 2 | 0.79788 | 9 | 0.96931 | 16 | 0.98348 |
| 3 | 0.88623 | 10 | 0.97266 | 17 | 0.98451 |
| 4 | 0.92132 | 11 | 0.96535 | 18 | 0.98541 |
| 5 | 0.93999 | 12 | 0.97756 | 19 | 0.98621 |
| 6 | 0.95153 | 13 | 0.97941 | 20 | 0.98693 |
| 7 | 0.95937 | 14 | 0.98097 |  |  |
| 8 | 0.96503 | 15 | 0.98232 |  |  |
